# Polypyrrole nanoparticles-based soft actuator for artificial muscle applications†

**DOI:** 10.1039/c9ra06900c

**Published:** 2019-12-02

**Authors:** Ajahar Khan, Khalid A. Alamry, Ravi Kant Jain

**Affiliations:** Faculty of Science, Department of Chemistry, King Abdulaziz University Jeddah 21589 Saudi Arabia; Microrobotics Laboratory/Information Technology Group, CSIR-Central Mechanical Engineering Research Institute (CMERI) Durgapur 713209 India kaalamri@kau.edu.sa arkhan.029@gmail.com

## Abstract

Currently, a straightforward fabrication technique for the development of soft actuators to explore their potential in robotic applications using environmentally compatible raw materials represents an important challenge. A conventional conducting polymer, such as polypyrrole (PPy), shows promising conductivity for such applications. This study presents the synthesis of PPy/polyvinyl alcohol (PPy/PVA)-based ion exchange polymer films containing PEDOT:PSS/SWNT/IL electrodes that undergo conformational changes in response to the applied voltage. Four types of ionic polymer actuator films with different sizes of PPy nanoparticles were fabricated to investigate the size-dependent electromechanical actuation performance. The aim of this study is to design and develop a stable, flexible, and reliable film actuator for robotic applications. Scanning electron microscopy and transmittance electron microscopy were performed to observe the surface morphology and detailed structure of the fabricated actuator films. The current density and ionic conductivity are demonstrated by the cyclic voltammogram and linear sweep voltammogram, respectively. The enhanced values of the water uptake, ion exchange capacity and ionic conductivity of the PPy/PVA polymer composite films enhanced the electrical properties and the tip deflection performance as compared to those of the other reported expensive perfluorinated polymer-based membrane actuators. A two finger-based micro gripping device was also developed, in which both the fingers were made up of the O-PPy/PVA/EL-based ion exchange polymer films. This mechanically stable and flexible film actuator fabricated *via* a synergistic combination of PPy/PVA composition containing PEDOT:PSS/SWNT/IL electrode surfaces possesses a substantial potential as an actuator material for micro robotic applications.

## Abstract 2

Currently, a straightforward fabrication technique for the development of soft actuators to explore their potential in robotic applications using environmentally compatible raw materials represents an important challenge.

## Graphical abstract

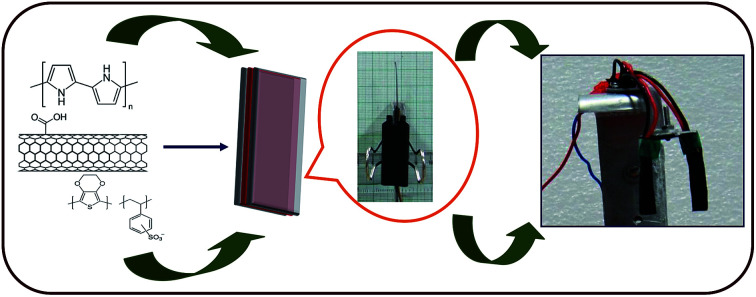

## 1. Introduction

For the last two decades, devices, such as soft actuators are sufficiently flexible to permit the relaxation and contraction on command in a wide range of movement patterns, are expected to mimick the motility of biological systems. This particular case of actuation led to the development of material-based artificial muscles and devices, which are encouraged by the mechanisms used by animals to propel on the ground or underwater.^1^ Recently, actuating devices that are competent to responding to external stimuli, such as light irradiation, electricity,^2,3^ moisture,^4^ IR and NIR light,^5^ temperature and humidity,^6^ UV light and heat,^7^ solvent concentration,^8^ and organic solvent type,^9^ have been developed. The intrinsic characteristics of electro-active polymer (EAP)-based ionic polymer soft actuators that offered the advantages of flexibility, lightweight, low power requirements, response to different stimuli, and being able to be processed at nanoscale expanded the realm of their promising applications.^10–12^ Therefore, EAPs have been widely used as dynamic component materials for the development of soft actuators because of their excellent properties to overcome the application problems of the electronic systems. A variety of EAPs, such as ionic polymer metal composites (IPMCs), conductive polymers, ionic piezoelectric polymers and polymer gels, have been widely used in the manufacture of artificial muscles and microrobots.^11,13,14^ Among the various types of EAPs, IPMC containing an electrolyte film sandwiched between the two platinum electrode layers has been widely used for the fabrication of actuators.^15^ Flexible composite materials and conducting polymer-based IPMCs generate a wide range of innovatory functional devices, including actuators, robots, and sensors that are curved, bendable and even stretchable.^16–22^ Typically, the common configuration of an EAP-based actuator consists of bi- or multilayer, in which the conductive electrode material is deposited or attached to an electroactive ion exchange polymer composite material substrate.^23–25^ The main rationale of the substrate is to mechanically counteract the EAP volume changes and form a stress gradient in the whole assembly structure under an external stimuli. This leads to the displacement or deformation through bending.^26,27^ Response to different stimuli, low power requirements and flexibility make the conductive polymers (CPs) functional constituent materials for the development of soft actuators.^28,29^ Polypyrrole (PPy) is one such CP possessing high strain and stress and high electrochemo–mechanical activity, under assorted applied stimuli, that has been extensively studied for the development of the actuators as well as sensors.^30–36^ Moreover, the biocompatibility of PPy makes it an interesting choice for the biomedical applications.^37–40^ PPy undergoes a rapid deformation or contraction due to the sorption or desorption of water vapors caused by the Joule heating, an effect induced under an applied voltage.^29,41^ An enhanced displacement, bending angle and response time of the fabricated devices can be achieved by using ion exchange polymer membranes, fibres or tubes^30,42^ that can assist the ion diffusion in the PPy films. PPy altered its volume as ions entered or left its structure *via* a diffusive course of action that is strongly dependent on the surface area.^43,44^ A tri-layer configuration consisting of two PPy layers separated by an electrolyte ion exchange polymer membrane^45–48^ or a solid ion intermediate platform^49,50^ can be operated in a dry condition. Moreover, with the low-cost raw materials and by using simple chemical and electrochemical methods, PPy can be easily obtained with different morphologies and configurations.^51^ Many researchers have paid attention to the efforts on improving the properties of electrode materials as the performance of the soft actuators are determined by the electrode materials. Therefore, due to the low cost, high electrical conductivity, environmental friendliness in doped states, and the remarkable storage capacity, PPy or poly(3,4-ethylenedioxythiophene):poly(4-styrenesulfonic acid) (PEDOT:PSS) is receiving a specific attention to serve as an electrode material.^52,53^ In particular, PEDOT:PSS has been extensively studied as an electrode material in dye-sensitized solar cells,^54–56^ organic photovoltaics,^57,58^ organic light-emitting diodes,^59^ and sensors.^60,61^ PEDOT:PSS has numerous advantages, including a small band gap (1.6–1.7 eV), good optical properties, high processability from aqueous solution, and a low redox potential.^57,62,63^ PEDOT is considered to be the widely used CP with regard to processability, electrical conductivity, and stability.^64^ While the PEDOT:PSS is found to be water soluble, it is commercially available as an aqueous dispersion with different conductivity grades. PEDOT:PSS can be used to develop uniform thin films by using various film forming techniques. Moreover, this material has a superior thermal stability and electrical conductivity (0.1–3000 S cm^−1^).^65,66^ Therefore, it has been employed for the fabrication of electrodes for actuators,^67,68^ and the conversion of electrical to mechanical energy has been verified by using these devices.^69,70^ It is generally assumed that the reduction in the size of the electrode material to the nanoscale level is an important approach to encourage its electrochemical performance. The nanosize dimension effect mainly aids in a shorter ion diffusion length and in achieving a higher surface area.^71,72^

In this study, we introduced a novel strategy to fabricate the electric stimuli responsive soft actuator by developing PPy-based ion exchange electrolyte films using PEDOT:PSS/SWNT/IL composite electrode, without using an expensive and time-consuming Pt metal electrode method. A series of experiments with various ratios of FeCl_3_ were designed and conducted to polymerize the pyrrole monomers. The results presented here revealed that the concentration of the oxidizing agents to polymerize the pyrroles could significantly improve the actuation performance and the mechanical strength of the developed actuator film, which might open up new strategies for further design and application of the advanced polymer-based materials for the micro robotics and biomedical engineering devices. FeCl_3_ was used as an oxidizing agent to initiate the chemical oxidative polymerization of pyrrole monomers, and polyvinyl alcohol (PVA) acted as a structure forming agent and as a stabilizer during the polymerization process.^73^ PVA and PEDOT:PSS are excellent water-soluble matrix polymers for forming hydrogels, which as individuals were not suitable for producing a durable actuator film and might not be suggested for robotic applications. The synergistic combination of conducting polymer PPy and cost-effective PVA (bio-compatible and with excellent film-forming capability) provided a mechanically stable and conductive ion exchange polymer film. The main role of PPy is to enhance the actuator performance by increasing the electrical conductivity of the actuator film under an applied low voltage and enabling the fabricated actuator film to be stable under both the double distilled water and the conventional electrolytes at an elevated temperature. Consequently, the presence of a highly conductive and flexible PPy film provided an additional support to the surface electrode and prevented it from surface cracking so that it could be free from the leakage of electrolytes or ionic liquids during the continuous actuation under an applied potential. In order to enhance the mechanical strength, electrical properties and durability of the electrode film, PEDOT:PSS was composited with SWNT. It was exceedingly desirable to add/mix PEDOT:PSS with an additional additive, such as carbon nanotubes, to enhance the mechanical rigidity and conductivity for the better performance of the fabricated membrane actuators. The single-walled carbon nanotube was chemically modified, which not only brought these structures into the fold of the (macro) molecular chemistry but also afforded ultimate new uniqueness that had attracted considerable attention. Moreover, the modification of SWNT could form a very stable dispersion, which was essential to form a uniform distribution of SWNT when compositing with PEDOT:PSS, PVA and an ionic liquid (IL). The addition of SWNT enhanced the mechanical strength and electromechanical properties of the proposed actuator film. PEDOT:PSS generated a hydrogen bonding and conjugate effect with the modified SWNT, which enhanced the electrical conductivity, altered the structural morphology and enhanced the surface area for the chemical reaction. Moreover, PEDOT:PSS exhibited an excellent film forming capability and contained sulfonic groups, which increased the available sites for proton exchange, WU and was also responsible for a high IEC. The combination of a large surface area and electrical conductivity of the individual SWNT nano-particles and excellent electrochemical properties of PPy and PEDOT:PSS composite materials provided a good physical–chemical properties and actuation performance. The presence of SWNT enabled the membrane actuator to be stable under both double-distilled water and the conventional electrolytes at room temperature (RT). In addition, it yielded a compact, uniform, brick and mortar structure. It was demonstrated by Iijima *et al.*^74^ that the combination of carbon nanotubes and PEDOT:PSS improved the chemical, mechanical and electrical properties.^75–77^ Terasawa *et al.* studied that the SWCNT-PEDOT:PSS-IL-based actuator exhibited an improved actuation performance and more superior electrochemical characteristics compared with that of PEDOT:PSS-IL actuator.^78^ Most of the ionic liquids (ILs) are liquids under ambient conditions and could be used as electrolytes in electrochemical systems and devices. They are characterized by their high ionic conductivities, which are likely to be favorable for large bending deformation of IPMC in open air. IPMC and CP-based actuators with ILs as electrolytes have already been reported by several research groups.^79–81^ Asaka *et al.* reported on the utilization of a bucky-gel (gel-like material) material to develop the first-ever dry actuator containing single-walled carbon nanotubes and IL.^82,83^ The combination of the individual properties of the PEDOT:PSS and the modified SWNT nano-particles afforded the admirable electromechanical, electrochemical and good physical–chemical properties. It might be considered that the PEDOT:PSS/SWNT/IL composite electrode would be acquiescent to actuate the fabricated soft actuator film by providing an unconventional electronic pathway under an applied electric potential. The two finger-based micro-gripping device for dexterous handling was developed using the O-PPy/PVA/EL-based actuator films for micro-robotic applications. The successful demonstration of a micro-gripper revealed the potential of the O-PPy/PVA/EL soft actuators for the next-generation soft robotic devices. Therefore, the proposed composite materials could be promising alternatives for the replacement of the environmentally unfriendly, expensive perfluorinated Nafion-based IPMC actuator.

## 2. Experimentals

### 2.1. Materials and methods

Pyrrole (reagent grade, 98%, MW 67.09 g mol^−1^), 4-sulfophthalic acid (50 wt% solution in water, technical grade, MW 246.19 g mol^−1^), poly(3,4-ethylenedioxythiophene)-poly(styrenesulfonate) (PEDOT:PSS) (2.8 wt% dispersion in H_2_O and low-conductivity grade) and 1-ethyl-3-methylimidazolium tetrachloro aluminate, ≥95% ionic liquid (IL) (Sigma-Aldrich Chemie Pvt., Ltd.), single-walled carbon nanotubes min. 90%, OD: 1–2 nm, length: 5–30 μm (SRL, Pvt., Ltd, India), poly(vinyl alcohol) (PVA; MW ∼115 000, Loba Chemie Pvt., Ltd, India), ferric chloride hexahydrate (MW 270.30 g mol^−1^, Himedia Pvt., Ltd, India), nitric acid (HNO_3_), sodium nitrate (NaNO_3_) (MW 84.99 g mol^−1^), sodium hydroxide palette (MW 40 g mol^−1^) and ammonium hydroxide (NH_4_OH; 25%, Merck Specialties) were used as received without any further purification.

### 2.2. Preparation of PPy films

PPy-based ion exchange polymer electrolyte films were prepared using pyrrole, PVA and FeCl_3_ as source materials in double-distilled water. First, 4 g PVA was dissolved in 40 mL double-distilled water under continuous stirring for 5 h at 70 °C. The viscous PVA solution was divided into 4 culture tubes, each containing a 10 mL volume. After complete dissolution, FeCl_3_ was mixed into all the four culture tubes containing the PVA solution under constant stirring for 1 h. The color of the solution mixtures evolved from a limpid state to a viscous orange. The concentration of FeCl_3_ varied in the range of 0.25, 0.50, 0.75 and 1.0 g relative to the 10 mL aqueous solution of PVA. Immediately, the *in situ* oxidative polymerization of the pyrrole monomer was performed by the drop-wise addition of 1 mL pyrrole monomer solution into all of the four culture tubes under constant stirring up to 1.5 h. As the polymerization proceeded the color of the solution mixtures changed from orange to black. After the completion of polymerization, the resulting solutions of polypyrrole-PVA (PPy/PVA) were maintained for 24 h at room temperature (RT) (25 ± 3 °C) for digestion. Herein, the concentrations of the pyrrole monomer and the PVA solution were kept constant. The concentration of FeCl_3_ was varied in the range of 0.25 to 1.0 g (ESI Table S1†). Hence, all the four fabricated samples based on 0.25, 0.50, 0.75 and 1.0 mL of FeCl_3_ concentrations were named as O-PPy/PVA, P-PPy/PVA, Q-PPy/PVA and R-PPy/PVA, respectively. Finally, 5 mL of each solution mixture was casted into a Petri dish (100 × 15 mm [S line]) and covered with a porous aluminum foil. The Petri dish was kept in a hot air oven at 60 °C for 10 h. After the complete evaporation of the solvents, the as-prepared films were thermally treated at 120 °C for 1.5 h. The dried O-PPy/PVA, P-PPy/PVA, Q-PPy/PVA and R-PPy/PVA films (0.19 ± 0.007 mm thickness) were washed with double-distilled water and acetone several times and then dried at 60 °C before being sandwiched between the electrode films.

### 2.3. Preparation of electrode film

The fabrication of the electrode films involved two main steps: in the first step, commercially obtained single-walled carbon nanotubes were converted into an acidic form by sonicating in 1 : 3 (v/v) concentrated nitric and sulfuric acids for 5 h at 50 °C. The resultant solution suspension was washed thoroughly using double-distilled water until neutralization (pH 6–7). Then, the obtained acid form of SWNT-COOH (SWNT) was collected and dried at 60 °C in a hot air oven. A homogenous dispersion was formed by adding 40 mg of the SWNT powder into 10 mL double-distilled water under a strong mechanical agitation (2 h), followed by sonication for 1 h at RT. In the second step, a mixture of 5 mL PEDOT:PSS (2.8 wt% dispersion in H_2_O) and 5 mL of the as-prepared SWNT dispersion was made in a culture tube under constant stirring for 3 h at 50 °C. Immediately, 7 mL of the aqueous solution of PVA (1 g dissolved in 10 mL double-distilled water) and 40 mg IL was added to the above solution mixture under constant stirring for 5 h at 50 °C. Then, the solution mixture of the PEDOT:PSS/SWNT/IL electrode was degassed by sonicating for 1.5 h at 45 °C. Finally, 3 mL of the obtained homogenous viscous solution was then casted onto a clean glass slide and kept in a hot air oven at 50 °C. After drying, the electrode films were removed from the glass slide and washed with acetone. Then, the as-prepared electrode films (with 0.12 mm thickness) were transferred into a hot air oven at 80 °C and dried overnight to remove the acetone residue.

Finally, the fabrication of the soft actuator film was carried out by sandwiching the as-prepared O-, P-, Q- and R-PPy/PVA ion exchange polymer films with the developed electrode films PEDOT:PSS/SWNT/IL using a hot press. The thicknesses of the final actuator films were less than the sum of the thicknesses of their constituent films because each membrane became thin owing to hot pressing. Finally, after sandwiching between the electrodes, the actuator films termed as O-PPy/PVA/EL, P-PPy/PVA/EL, Q-PPy/PVA/EL and R-PPy/PVA/EL were treated with a 0.2 M NaOH aqueous solution at RT for 2 h for the exchange of metal cations and used for further characterizations.

### 2.4. Characterization

The as-prepared ion exchange polymer films of PPy before and after the PEDOT:PSS/SWNT/IL electrode layer deposition were morphologically characterized *via* field emission-scanning electron microscopy (FE-SEM) (Make: Zeiss, Germany) and transmission electron microscopy (TEM) (TEM Jeol, JEM-2100, Japan), respectively. The confirmation of the chemical constitution and structure was done by recording the FTIR spectra under a working range of 4000–500 cm^−1^ on a Perkin Elmer-made FT-IR spectrum 100 spectrometer. The electrical properties of the O-, P-, Q- and R-PPy/PVA/EL films were measured on a modular potentiostat/galvanostat (Autolab 302N) associated with an impedance analyser (FRA32M.X). To investigate the electrical parameters, cyclic voltammetry (CV) at ±2 V, linear sweep voltammetry (LSV) at 0–2 V under a scanning rate of 100 mV s^−1^ and ionic conductivity or proton conductivity (PC) at a frequency range of 100 KHz under an AC annoyance of 100 mV s^−1^ were recorded in a 0.1 M NaOH aqueous solution, which was used as an electrolyte. A customary three-electrode framework, including the O-, P-, Q- and R-PPy/PVA/EL-based actuator films, was used as the working electrode. Ag/AgCl used as the reference electrode and a platinum wire used as the counter electrode were utilized for CV, LSV and PC investigations. The tensile properties of all the four actuator films were studied with the help of a universal testing machine (model: H50 KS, Shimadzu Corp.). Bending behaviors of the fabricated actuator films were determined using a laser displacement sensor (model: OADM 20S4460/S14F; Baumer Electric, Germany) at 0–2 V DC through a computer-controlled digital analogue card (DAC) and a micro controller. The tip deflection error was controlled by using a proportional integral derivative (PID) control system by tuning the frequency to set the controller bandwidth. A digital weighing/load cell (model: Citizen CX-220, make: India) was used to demonstrate the load characterization. After characterizations, a compliant two finger-based micro-gripping system with a dedicated control system was developed using O-PPy/PVA/EL actuator films.

## 3. Results and discussion

The size proscribed PPy nanoparticle-based ion exchange polymer membrane was synthesized on the basis of the complexes formed between the Fe^3+^ cations and the PVA in order to initiate the oxidative polymerization of the pyrrole monomers. A series of experiments are designed and conducted with a fixed ratio of PVA and pyrrole and by varying the ratio of FeCl_3_, as shown in ESI Table S1.† Here, the water soluble PVA acted as the stabilizer and as the structure forming agent, while FeCl_3_ acted as the oxidizing agent for initiating the chemical oxidative polymerization. As the pyrrole monomers came in contact with the Fe^3+^ cations in the aqueous dispersion of Fe^3+^/PVA complex, the polymerization started.^73^ The amount of the oxidizing agent (Fe^3+^ cations) utilized played an important role in the morphological structure of the polymer membranes. The results obtained from the fabricated polymer membranes revealed that the size of the polymerized PPy nanoparticles increased with an increase in the concentration of FeCl_3_. The electrostatic repulsion between the Fe^3+^ cations bound onto the PVA chain altered the conformation of the Fe^3+^ cations/PVA complex.^73^ At a lower concentration of Fe^3+^ cations, the electrostatic repulsion within the complex retained a condensed globular conformation, which permitted the enhanced particle firmness against further growth. Therefore, at lower concentrations of Fe^3+^ cations, smaller nanoparticles of PPy were obtained. Moreover, with an increase in the concentration of the Fe^3+^ cations, tough electrostatic repulsion between the complex of PVA/Fe^3+^ cations might lead to less control and conformational change within the complex, which resulted in large PPy nanoparticles.

### 3.1. SEM analysis

**Fig. 1:**
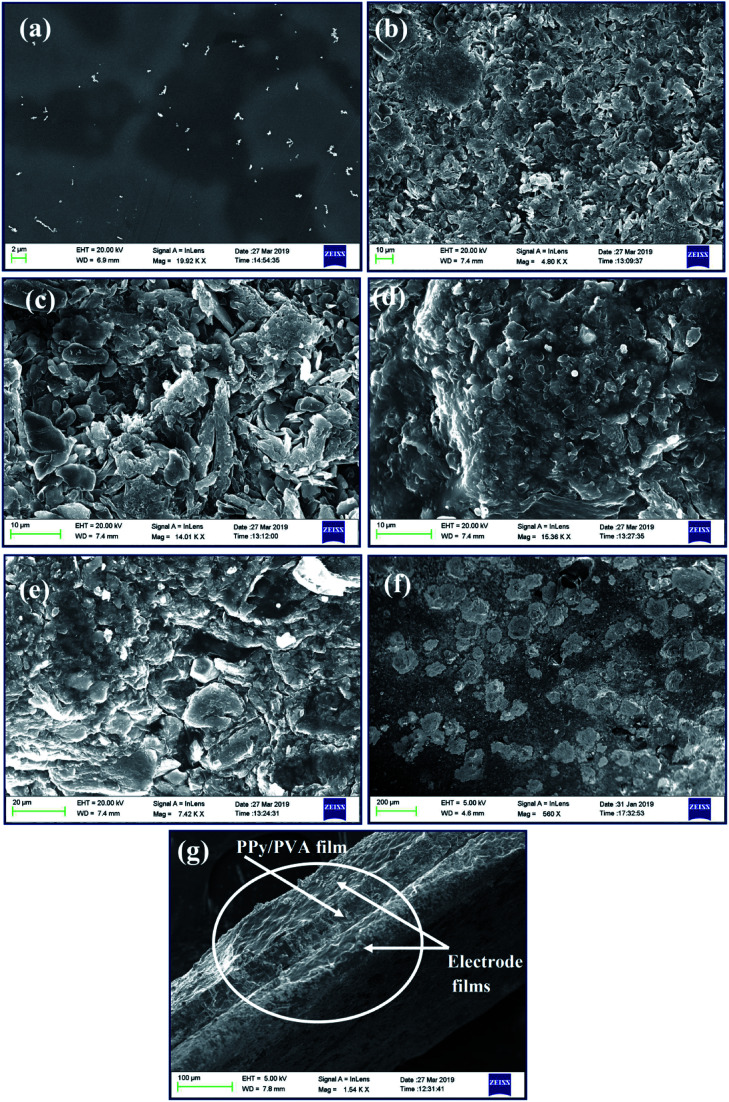
Surface morphology of (a) PVA film, (b–e) O-PPy/PVA, P-PPy/PVA, Q-PPy/PVA and R-PPy/PVA ion exchange polymer films, (f) PEDOT:PSS/SWNT/IL (EL)-based electrode film and (g) cross-sectional view showing the electrolyte polymer film sandwiched between the electrodes films.

The morphology of the as-prepared ion exchange polymer films of PPy/PVA before and after the PEDOT:PSS/SWNT/IL electrode layer deposition is observed *via* FE-SEM as shown in Fig. 1 and *via* TEM as shown in ESI S.1† (TEM). The FE-SEM micrographs (Fig. 1(a)) show that the pristine PVA films had a uniform smooth surface without any fracture and evident inner channels. However, the oxidative polymerization of pyrrole in the PVA solution led to a porous structure formed by the interpenetration of the PPy nanoparticles with different appearances (Fig. 1(b–e)). Fig. 1(b–e) demonstrates the surface morphology of O-PPy/PVA, P-PPy/PVA, Q-PPy/PVA and R-PPy/PVA ion exchange polymer films, respectively, in which the PPy nanoparticles crystallized into many irregular polymer grains along with porosities and inner channels in their free spaces for the IL migration. This rough structure with polymer grains also enhanced the surface interactions with the electrode membrane and conductivity of the ion exchange polymer actuator, which can be projected to result in higher actuation strokes.^84^Fig. 1(b) shows that at low concentration of FeCl_3_ the deposition of PPy nanoparticles on PVA leads to the fine polymer grains containing surface structure of the fabricated films with smaller PPy nanoparticles. Consequently, as the concentration of FeCl_3_ increased to polymerize the pyrrole monomer as shown in Fig. 1(c–e), strong electrostatic repulsion among the complex of PVA/Fe^3+^ cations may lead to the less control and conformational change within the complex, which results in large PPy nanoparticles. Moreover, the electrode films containing PEDOT:PSS/SWNT/IL are shown in Fig. 1(f). The electrode film shows a network of irregular macroscale and some flaky structures appearing at the surface and the grain boundary free from surface crack and porosity. Subsequently, the deposition of SWNT on the surfaces of PEDOT:PSS displays a wavy and wrinkled morphology (Fig. 1(f)) due to the intercalations of oxygen-related functionalities. Fig. 1(f) clearly shows the snow-like appearance distributed throughout the surface of the electrode film. Since, PEDOT:PSS has conjugated π bonds, the interactions with the modified SWNT should be strong due to π–π stacking and van der Waals force of attractions. These attractions play a significant role in the sticking of the polymer films and transferring of the force from one layer to another layer. As the fabricated PPy/PVA-based electrolyte polymer films was sandwiched between the electrode films, the surface cracks and porosity were no longer observed, except the irregular macroscale polymer grains and some flaky structures. Fig. 1(g) clearly shows the cross-sectional view of the fabricated film actuator. In this figure, the intermediate film shows the PPy/PVA ion-exchange polymer electrolyte film, which is sandwiched between the two PEDOT:PSS/SWNT/IL-based electrode films.

### 3.2. The solvent uptake property and ion exchange capacity

**Fig. 2:**
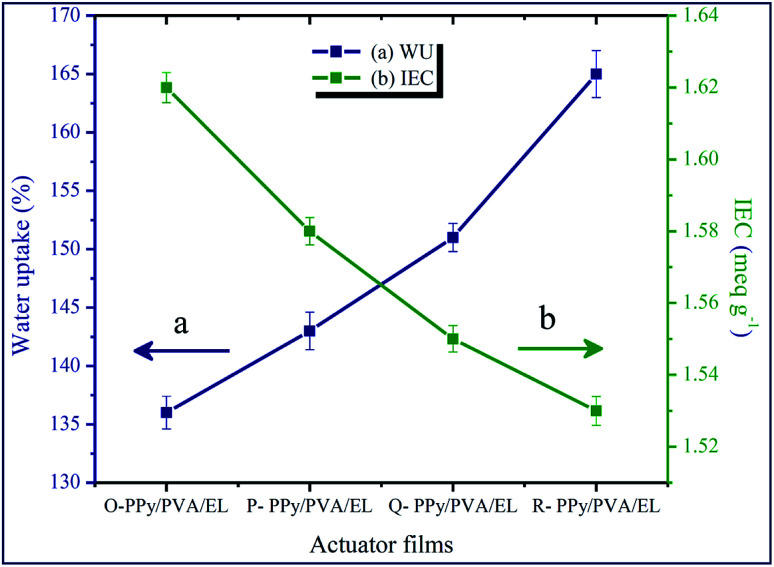
(a) Water uptake and (b) IEC for the fabricated ionic polymer film actuators.

Water uptake (WU) or the holding behavior and IEC are two vital properties of the ionic polymer membrane-based soft actuator. A combination of PEDOT:PSS-based electrode and PPy-based ionic polymer film was used as the ionic conducting material in the proposed soft actuator, and it had a better ionic conductivity, which came primarily from its sulfonic acid groups and from the conductive behavior of PPy in its structure. The movement of the hydrated cations in the inner solvent, such as water molecules, under an electrical stimulus was the foremost cause of the bending of a film actuator. The high WU deteriorated the mechanical stability of IPMCs, while it improved the dielectric constant and proton conductivity or ionic conductivity.^85,86^ Hence, the WU of the fabricated film actuators should be in an adequate amount for the voyage of the hydrated metal cations. The WU of O-PPy/PVA/EL, P-PPy/PVA/EL, Q-PPy/PVA/EL and R-PPy/PVA/EL films was evaluated by the difference between the totally hydrated and dried out mass of the films. To determine the WU, the actuator films were immersed in an excess of double-distilled water at RT for 24 h. The WU analysis suggested that as the concentration of the Fe^3+^ cations increased from O-PPy/SP to R-PPy/SP the water holding capability increased. It was found that the O-PPy/PVA/EL showed a minimum WU, *i.e.* 136% in comparison to the P-, Q- and R-PPy/PVA/EL films, which had a WU of 143%, 151% and 165%, respectively (Fig. 2(a)). The reason for the increase in the WU from O-PPy/PVA/EL to R-PPy/PVA/EL was that the size of the polymerized PPy nanoparticles within the fabricated membranes increased with an increase in the concentration of FeCl_3_. Therefore, with an increase in the particle size of PPy, the pore size between the polymer grains in the fabricated polymer films increased, which allowed more water molecules to be trapped and absorbed within the porous structure. This led to the increase in the WU capacity from O-PPy/PVA/EL to R-PPy/PVA/EL membranes and found to be the main cause of variation in the WU behavior of the proposed compositions. Subsequently, it was observed that the water holding capability of O-PPy/PVA/EL was much more adequate to initiate the migration of hydrated than other reported IPMC actuator membranes due to the high IEC (Fig. 2(a)).^15^

The IEC values for all the four fabricated membrane actuators are also determined and plotted in Fig. 2(b). The result obtained from the IEC analysis showed that the IEC values also decreased with an increase in the Fe cations to the polymerized PPy from O-PPy/PVA/EL to R-PPy/PVA/EL membranes. At a high concentration of FeCl_3_, electrostatic repulsion between the Fe^3+^ cations bound on the PVA chain altered the conformation of the Fe^3+^ cations/PVA complex.^73^ Therefore, as we moved from O-PPy/PVA/EL to R-PPy/PVA/EL, the large sized polymer grains of the polymerized PPy within the fabricated membrane actuators decreased the mechanical firmness and compactness. Moreover, at a low concentration of FeCl_3_, the small-sized polymer grains of the polymerized PPy improved the mechanical stability of the fabricated ionic polymer membranes and also helped to attain the hydrophilic volume in an adequate amount as well as the availability of more sulfonic acid groups. Therefore, the maximum IEC was found to be 1.62 meq g^−1^ for O-PPy/PVA/EL in comparison to the P-, Q- and R-PPy/PVA/EL membranes, which had IECs of 1.58, 1.55 and 1.53 meq g^−1^, respectively. Thus, an adequate amount of the WU capability and the higher IEC meant that more water molecules moved towards the negative electrode by the stroke of hydrated cations. Electrophoresis and electro-osmosis caused more volume expansion in the direction of the cathode, which led to the better actuation performance of the fabricated membrane actuator.^87,88^ The high ionic or proton conductivity enabled the better actuation performance and durability of the ionic polymer film actuators. Implantations of smaller PPy particles enhanced both the electric and proton conductivities, decreased the resistance, and improved the electro-mechanical exchange efficiency of the fabricated O-PPy/PVA/EL actuator film. The proton conductivity of O-, P-, Q- and R-PPy/PVA/EL-based actuator films was found to be 4.124 × 10^−2^, 4.102 × 10^−2^, 3.861 × 10^−2^ and 3.812 × 10^−2^ S cm^−1^, respectively. It could be clearly observed that with a decrease in the PPy particle size of the fabricated actuator films, the ionic conductivity increased and found to be at maximum for O-PPy/PVA/EL because of the uniformly distributed small polymer granules of PPy nanoparticles, which increased the hydrated cations for the migration towards negative electrodes. The high proton conductivity created strong electrostatic interactions between the negative electrode and movable cations, which caused more cations to migrate towards the negative electrodes under an applied electrical potential. Therefore, the high ionic or proton conductivity enabled the more hydrated cations to move fast toward the cathode side to create an uneven pressure in the inner side and display a large displacement and fast actuation.^15,89^ Thus, the rate of the tip deflection of an actuator film enhanced due to the high proton conductivity or ionic conductivity. In the present study of IEC, ionic conductivity along with the WU (Fig. 2) for the fabricated O-PPy/PVA/EL membrane actuator confirmed that the proposed actuator had a higher ionic content or ionic conductivity when compared to a Nafion-based IPMC actuator.^15^ Therefore, it was supposed that O-PPy/PVA/EL-based membrane actuator attributed to a more compact structure with an adequate water holding capability and showed a better actuation performance than the remaining membrane actuators.

### 3.3. Mechanical properties

**Fig. 3:**
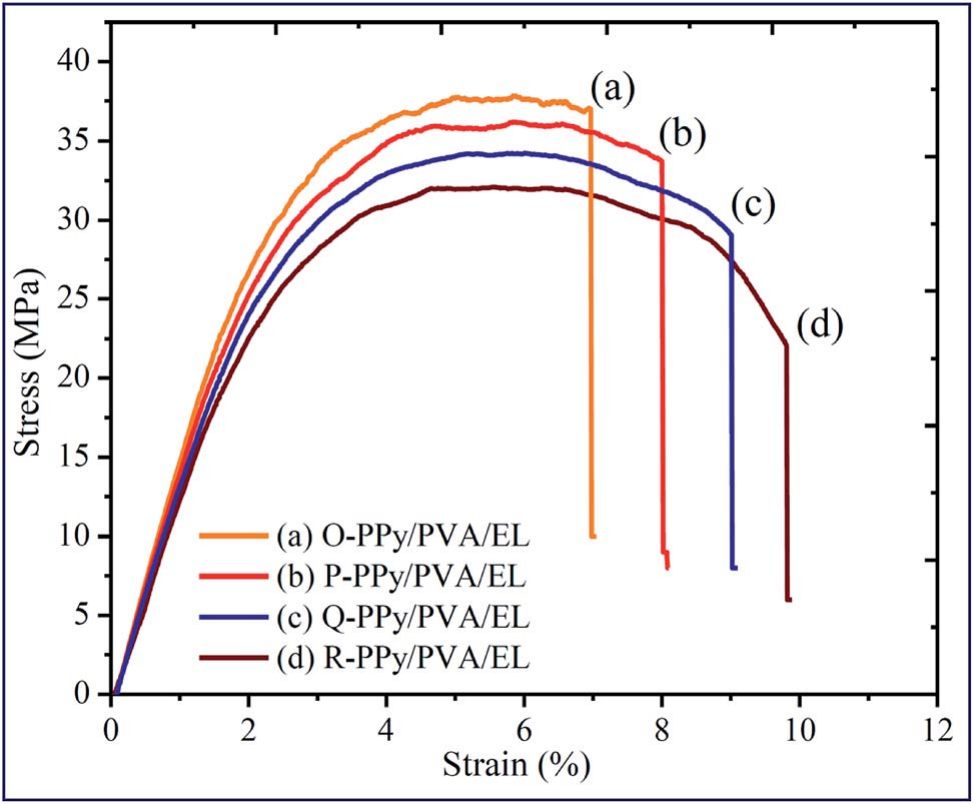
Tensile properties of (a) O-PPy/PVA/EL, (b) P-PPy/PVA/EL, (c) Q-PPy/PVA/EL and (d) R-PPy/PVA/EL-based film actuators.

The Young's modulus as well as the tensile strengths of the ionic polymer actuator films strongly influenced the actuation stiffness, the fabrication parameters, and stability in developing actuator films.^90,91^ The tensile strain of an actuator film was correlated with the thickness, the tip deflection and length of the ionic polymer film actuator.^92^ If the fabricated actuator films were not mechanically stable, then after performing a few actuation tests the cracks would develop on the surface of the film and damage it to some extent and make it unstable for further use. Therefore, the mechanical properties were important for the long-term bending deformation of the ionic polymer actuator films. The large tensile strengths and strains provided more flexibility and stability, which were important to provide a long-term actuation.^93^ The presence of SWNT and PPy provided mechanical strength and impact resistance to support the generation of the strong tip-generated force, whereas the PVA and PEDOT:PSS offered flexibility and benefitted the generation of a large tip deflection of the fabricated ionic polymer soft actuator. Fig. 3 shows the stress–strain curves of O-PPy/PVA/EL, P-PPy/PVA/EL, Q-PPy/PVA/EL and R-PPy/PVA/EL ion exchange polymer actuator films (size = 30 mm length, 10 mm width and 0.21 ± 0.002 mm thickness). All of the fabricated films exhibited a linear elastic region, followed by a nonlinear deformation. The Young's modulus, percentage elongation break, and ultimate tensile strength are calculated and reported in ESI Table S2.†Fig. 3 clearly shows that the ductility of the fabricated films increased as we moved from O-PPy/PVA/EL to R-PPy/PVA/EL. The tensile result showed that the R-PPy/PVA/EL actuator film was the most ductile, whereas O-PP/PVA/EL was somewhat less ductile with respect to the elongation break. The mechanical strength of the O-PPy/PVA/EL actuator film (Young's modulus = 568.41 MPa, ultimate tensile strength = 37.72 and MPa percentage elongation 6.93%) was more when compared to those of the others, so it could not take much load (ESI Table S2†). The tensile test analysis revealed that as the concentration of the oxidising agent increased to polymerize pyrrole into PPy from O-PPy/PVA/EL to R-PPy/PVA/EL, the Young's modulus and the ultimate tensile strength decreased. At a low concentration of FeCl_3_, the deposition of the PPy nanoparticles over the PVA *via* an *in situ* oxidative polymerization reaction was sufficient, which led to the formation of the fine PPy grains containing the surface structure (Fig. 3). These fine PPy polymer grains contributed to form a compact structure for the development of a mechanically strong ionic polymer actuator film (Fig. 1(b)). The improved mechanical strength of the O-PPy/PVA/EL ion exchange electrolyte actuator film was due to the fine PPy grain particles and the modified SWNT, which had superior electrical properties as well as excellent mechanical strength, which confirmed the proposed materials as alternatives for the development of ionic polymer actuators with better performance.

### 3.4. Electrical and electrochemical properties

**Fig. 4:**
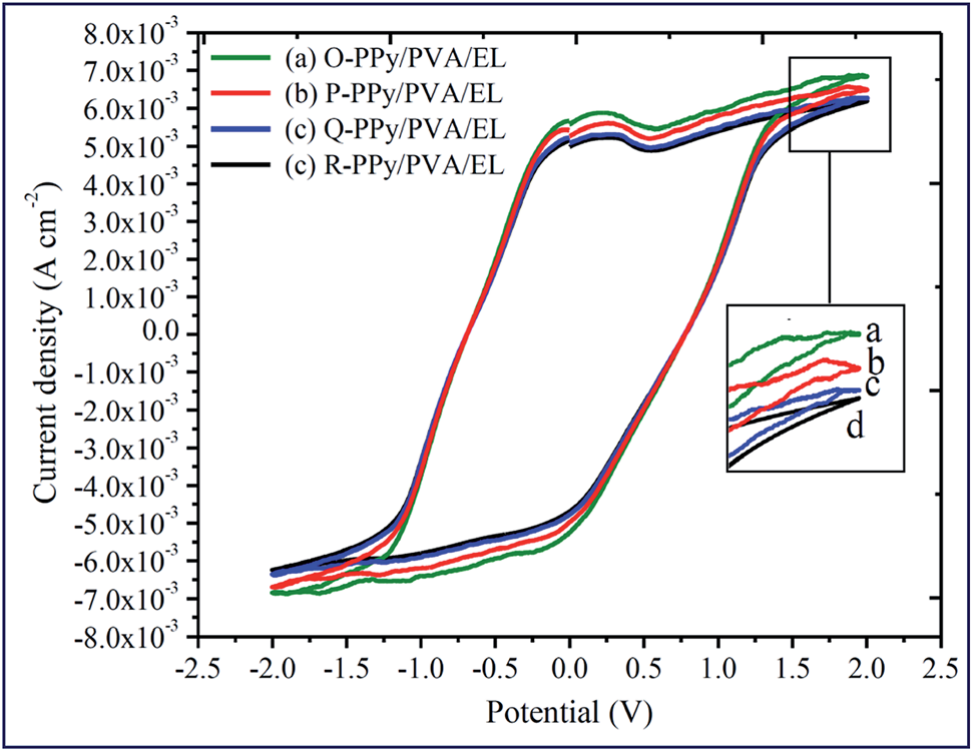
Cyclic voltammograms of (a) O-PPy/PVA/EL, (b) P-PPy/PVA/EL, (c) Q-PPy PVA/EL and (d) R-PPy/PVA/EL-based actuator films.

**Fig. 5:**
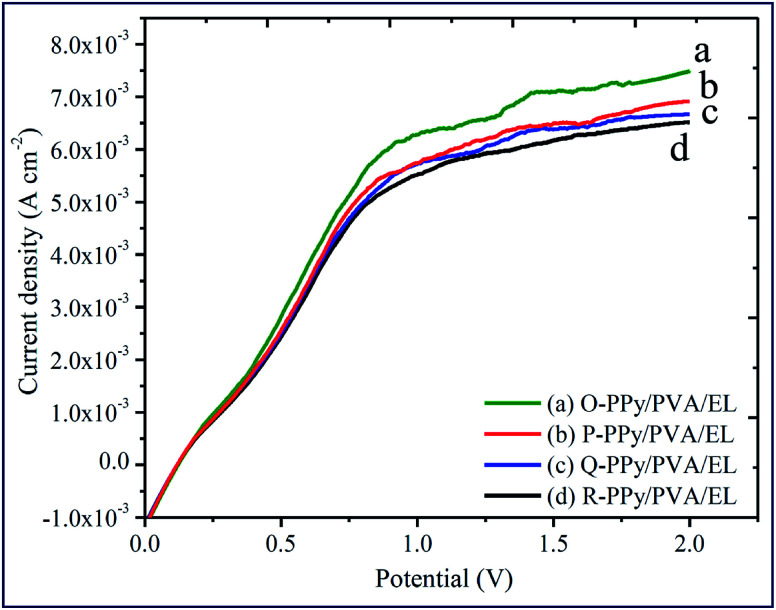
Linear sweep voltammograms of (a) O-PPy/PVA/EL, (b) P-PPy/PVA/EL, (c) Q-PPy PVA/EL and (d) R-PPy/PVA/EL-based actuator films.

The actuation performance of the actuator film was closely related to the electrochemical properties. The nature of the electrodes on the actuator films directly influenced the magnitude of the tip displacement and response time of ionic polymer actuators. The capability of the developed electrodes to exhibit a high current density or ionic conductivity mainly depended on the chemical affinity and the porous structure of the adopted electrode material (Fig. 1(f)). In this study, to evaluate the electrical properties of the fabricated polymer composite-based actuator films cyclic voltammogram (CV) and linear sweep voltammogram (LSV) were measured at the electrochemical window of ±2 V and 0–2 V, respectively, at a scan rate of 100 mV s^−1^ (Fig. 4 and 5). Fig. 4 shows the cyclic voltammograms of the fabricated O-, P-, Q-, and R-PPy/PVA/EL actuator films to analyze the consequence of the electrode on the electrochemical characteristic of the films. As can be observed, all of the cyclic voltammograms are approximately similar in shape but differed in the magnitude of the current. The symmetric shape and higher magnitude of the current density suggested that the electrode surface was providing an excellent charge distribution throughout the actuator film. As shown in Fig. 4, the broad shape of the *I*–*V* curves obtained for all the four fabricated films is indicative of the excellent capacitive behavior with a good ion migration response.^94^Fig. 4 also shows that despite the absence of any prominent redox peaks, the pseudocapacitive contribution and various sequential small peaks cannot be avoided. This was indicative of ionic diffusion in the proposed actuator films was chaotic, which may be ascribed to the rough and porous surface of the actuator films (Fig. 1(b) and (e)).^95,96^ The fabricated actuator films exhibited characteristic redox peaks at ∼0.3 V (Fig. 4 (a–d)), which corresponded to the oxidation and reduction reactions of PEDOT:PSS.^97^ The anodic peaks could be seen at the ∼0.3 V electrode potential without corresponding cathodic peaks, which indicated the reduction of the PEDOT:PSS at this potential. Therefore, the PEDOT:PSS electrode surface facilitated the electron transfer during the stimulation through an electrode–electrolyte junction. In Fig. 4, the peaks of PPy can be observed at 0.52 and 0.25 V, and these peaks may be due to the oxidation and reduction reactions of PPy in O-, P-, Q-, and R-PPy/PVA/EL actuator films.^98,99^ The area under the cyclic voltammogram obtained for O-PPy/PVA/EL was larger than that obtained for the P-, Q-, and R-PPy/PVA/EL actuator films (Fig. 4). This revealed that the redox behavior of PPy with smaller particles was improved by enhancing the interconnectivity among the PPy particles within the fabricated actuator film, which increased the current at the electrodes.^100,101^ Cyclic voltammograms suggested that the high magnitude of the current density of the actuator electrodes maintained a pseudo-constant rate over *I*–*V* cycles due to the electrosorption or intercalation on the electrode surface. The shape of the cyclic voltammogram reflected the movement of the cations under applied voltages, with a decomposition profile of the inner solvent molecules due to the electrolysis. The CV results suggested that the higher current density represented a high energy storage aptitude of O-PPy/PVA/EL (Fig. 4(a)), which indicated a large tip displacement and enhanced actuation performance under an applied electrical potential. It was clear from the LSV analysis that as the applied electric potential increased from 0 to 2 V, the current density of all the fabricated actuator films also increased constantly and found to be maximum for O-PPy/PVA/EL (Fig. 5). This might be due to the presence of a uniformly distributed small polymer granules of the PPy nanoparticles, the high ionic conductivity, IEC and adequate amount of the WU capacity. This enhanced the ionic transfer of the O-PPy/PVA/EL actuator film, which was essential to achieve a better actuation performance of the ionic polymer-based actuators.

### 3.5. Electromechanical properties

In order to investigate the electromechanical actuation behavior of O-PPy/PVA/EL, P-PPy/PVA/EL, Q-PPy/PVA/EL and R-PPy/PVA/EL ion exchange polymer actuators, the fabricated films were gripped in a cantilever configuration (ESI Fig. S3†). This cantilever configuration was connected to a digital power supply along with an NI-PXI system. The schematic representation and the actual view of the developed experimental setup are shown in ESI Fig. S3.† For activating the actuator films, the developed testing setup sent the preferred proscribed voltage (0–2.0 V) by means of a LabVIEW software with designed VI for controlling the voltage. To achieve the suitable tip deflection, an algorithm was also developed, where a PID control system feature enabled the incorporation of the PID parameters. To obtain the accurate tip displacement with respect to the applied potential for all the four fabricated film actuators, a laser displacement sensor was used. The second reason for using this displacement sensor was to acquire the displacement response for measuring and controlling the tip deflection. For all this in order to attain the proper communication between sensor and input command, the data conversion can take place by converting the data from RS-485 to RS-232 communication protocol interfaced with the NIPXI system and NI-VISA interface software module in LabVIEW VI. Moreover, at the same time, the DAC assistant of the NI-PXI system definite the deflection voltage from the programmable power supplies. The displacement sensor continuously monitored the tip displacement of the actuator films to attain the maximum tip deflection.

**Fig. 6:**
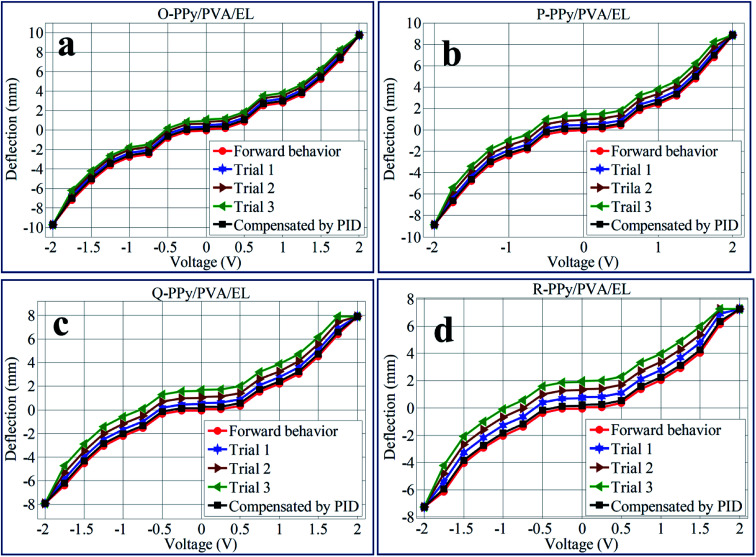
Tip deflection hysteresis behaviors of (a) O-PPy/PVA/EL, (b) P-PPy/PVA/EL, (c) Q-PPy/PVA/EL and (d) R-PPy/PVA/EL-based actuator films.

In the fabricated O-, P-, Q-, and R-PPy/PVA/EL actuator films, the SWNT-COO^−^ acted as polyions along with the negative charges. When the fabricated actuator film was subjected to applied electric voltages (DC), the cations of SWNT-COO^−^ and -SO_3_^−^ (in PEDOT:PSS) inside the actuator film migrated towards the negative electrode, while the polyions SWNT-COO^−^ and -SO_3_^−^, which were immobile remain fixed at their positions, which is similar to the actuation principle of typical IPMCs.^15^ After activation, the fabricated films bend and demonstrate a dexterous behavior. The successive stepwise deflection patterns of the O-PPy/PVA/EL film under an applied voltage of 0–2 V DC are shown in ESI Fig. S4.† To check the repeated actuation performance of all the four fabricated films, the same experiment at similar applied voltages was repeated for 4 × 6 (24) times. The obtained deflection values were collected at a voltage of 0–2 V DC for 24 times (provided in ESI Tables S3–S18†). The deflection mean values calculated from each set of 6 different observations (ESI Tables S3–S18†) were used as one trial to draw the deflection hysteresis plots (Fig. 6). This hysteresis analysis revealed that the increase in the voltage from 0 to 2 V resulted in the linear bending behavior for all the four types of fabricated actuator films, while reversing the voltage from 2.0 to 0 V DC, *i.e.* from high to low, the actuator films did not track the similar path to come back to its original position and showed some deflection error or hysteresis (Fig. 6(a–d)). To minimize this hysteresis or deflection error, the PID parameters were tuned into the LabVIEW software. This PID control system could minimize the deflection error by up to 80%. The results obtained from the hysteresis analysis suggested that by repeating the tip deflection experiments, the hysteresis curves for P-PPy/PVA/EL, Q-PPy/PVA/EL and R-PPy/PVA/EL were found to be broader in comparison with that obtained for the O-PPy/PVA/EL actuator film. The broader the surface area between the hysteresis curve, the higher would be the deflection error, as the actuator film deviated from its actual path or did not follow the same path to return to its original position while returning when the applied voltage was reduced from high to low. Hence, the broader shape of the hysteresis curve revealed the lower performance of the actuator films after multiple repetitions. Therefore, it was clear that with the repetition of the deflection experiments, the deflection error for P-PPy/PVA/EL, Q-PPy/PVA/EL and R-PPy/PVA/EL increased. Therefore, the performance of all the three films after the multiple repetitions was poor when compared to that of O-PPy/PVA/EL film. In case of O-PPy/PVA/EL with the repetition of deflection experiments, there was no significant effect on the hysteresis curve, which confirmed the better performance after the multiple repetitions. Thus, for the O-PPy/PVA/EL actuator film, an adequate amount of WU, ionic conductivity and higher IEC allowed more metal cations along with water molecules towards the negative electrode by the stroke of hydrated cations. Electrophoresis and electro-osmosis caused more volume expansion and led to the enhanced actuation performance.^87,88^ Therefore, the composition of the O-PPy/PVA/EL actuator film was suitable to show the enhanced actuation performance than rest of three compositions.

**Fig. 7:**
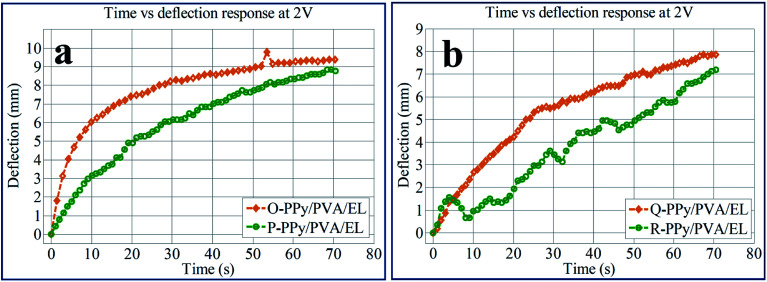
Time *vs.* deflection responses for (a) O-PPy/PVA/EL and P-PPy/PVA/EL, and (b) Q-PPy/PVA/EL and (d) OR-PPy/PVA/EL-based actuator films.

To analyse the bending response for all of the four fabricated actuator films, the tip movement with respect to time was carried out at 2 V DC, as shown in Fig. 7(a) and (b). From the time *vs.* tip displacement analysis, it was demonstrated that the tip displacement for all of the four O-PPy/PVA/EL, P-PPy/PVA/EL, Q-PPy/PVA/EL and R-PPy/PVA/EL actuator films increased up to 70 s under an applied 2 V DC. The results obtained suggested that the maximum tip displacement at 2 V DC after 70 s was found to be 9.85 mm for the O-PPy/PVA/EL-based actuator film. It is clearly shown in Fig. 7 that at 2 V DC, the tip deflection for all the actuator films increased but at the same time the behaviour of P-PPy/PVA/EL, Q-PPy/PVA/EL and R-PPy/PVA/EL films is slightly slow and irregular, while the O-PPy/PVA/EL actuator film showed a linear increase in the tip deflection. The analysis also confirmed that the large tip displacement, the precise bending behavior and overall performance of O-PPy/PVA/EL actuator film were much better than those of the other three compositions.

**Fig. 8:**
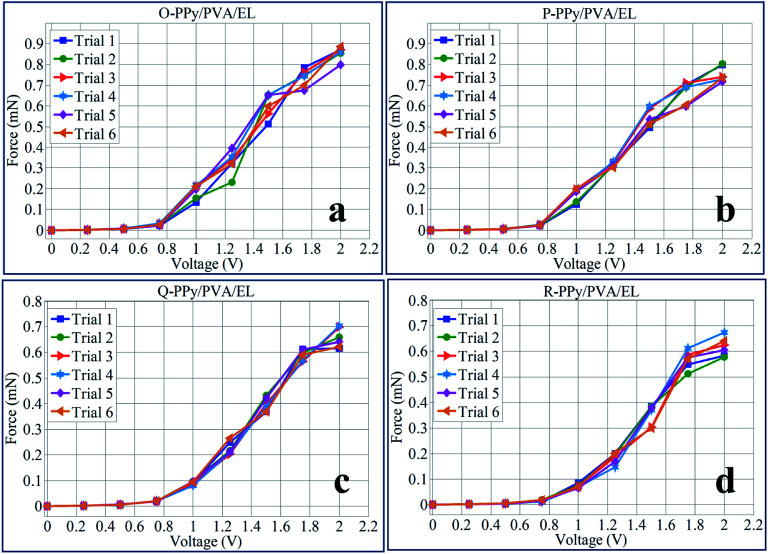
Generated tip force behavior at 0–2 V DC for (a) O-PPy/PVA/EL, (b) P-PPy/PVA/EL, (c) Q-PPy/PVA/EL and (d) R-PPy/PVA/EL-based actuator films.

In order to demonstrate the load characterization or force behavior of O-PPy/PVA/EL, P-PPy/PVA/EL, Q-PPy/PVA/EL and R-PPy/PVA/EL actuator films, a digital load cell was taken (load ranging from 0.0001 to 220 g). The load cell was positioned at the tip of the actuator film for measuring the tip load, as described in our previous study (ESI Fig. S5†).^14^ The results of the load characterization showed that the O-PPy/PVA/EL actuator film had the maximum tip generated force of 0.856 mN than four fabricated actuators at 2 V DC. At the same applied voltages, the tip generated force values for P-PPy/PVA/EL, Q-PPy/PVA/EL and R-PPy/PVA/EL actuator films were found to be 0.754, 0.656 and 0.617 mN, respectively. In order to demonstrate the tip generated force behavior after repetition, several trials of the same experiments for all the four fabricated films were conducted as F1, F2, F3, F4, F5 and F6, respectively (ESI Tables S19–S22†). The force mean values calculated from the six different observations obtained at different voltages were used to plot the force behavior under the varying voltages and to find out the standard deviation (ESI Tables S19–S22†). Fig. 8 shows that for all the 6 trials, the O-PPy/PVA/EL-based actuator film generated more force than the P-PPy/PVA/EL, Q-PPy/PVA/EL and R-PPy/PVA/EL-based actuator films. The load characterization analysis also revealed that as the concentration of the Fe^3+^ cations to the polymerized PPy in the fabricated films increased, the overall performance of the actuators decreased.

### 3.6. Two finger-based micro-gripper system

**Fig. 9:**
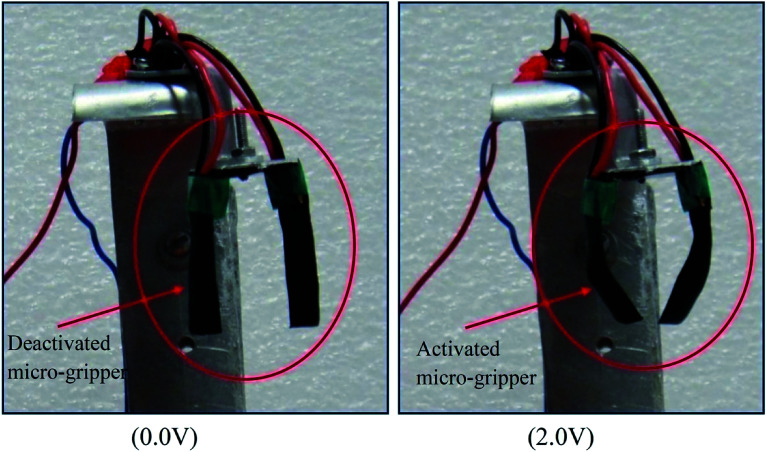
Two finger-based micro-gripping system developed using the O-PPy/PVA/EL-based actuator film.

After characterizing all the four fabricated ionic polymer actuator films, it was found that the O-PPy/PVA/EL-based actuator film showed an enhanced and better actuation performance. Therefore, the PPy/PVA/EL-based actuator films were used to develop a compliant two finger-based microgripper with a dedicated control system. In the developed microgripper, both the fingers were made up of O-PPy/PVA/EL films (size 30 mm length and 10 mm width) (Fig. 9). As shown in Fig. 9, the two O-PPy/PVA/EL film-based fingers were integrated in a wrist, which is clamped with a holder. The mimicking of the fingers was done *via* the electrical actuation instead of the conventional motor. For activating the actuator fingers, 2 V DC was provided through a PID controller. When the voltage was supplied through the controller, the micro-gripper becomes activated and both the fingers bend simultaneously toward the inner direction. Then, when the voltage was released or stopped the fingers were moving in a reverse manner and became deactivated. Here, each finger required an individual power supply to become activated and bent according to the given stimuli. Therefore, the handling of the two finger-based microgripper system was somewhat easier in comparison to the control three or four finger-based microgripper. The use of the O-PPy/PVA/EL-based actuator film as a two finger based microgripper system was demonstrated, which showed that the fabricated actuator film had the potential for developing artificial muscles and could also be used in the complex fabrication and assembly in robotics.

## 4. Conclusions

In this study, we developed novel ionic polymer composite-based soft actuator films with varying particle sizes of PPy nanoparticles. The PPy/PVA ionic polymer nanocomposite actuator films with PEDOT:PSS/SWNT/IL electrodes had improved tip deflection and mechanical strength in comparison to other reported IPMCs that had been demonstrated successfully. Using a film-casting method, a new hybrid-type PEDOT:PSS/SWNT/IL-based electrode film was developed as a substitute for an expensive platinum metal used to coat the IPMC film by an electroless plating method. Four types of ionic polymer actuator films, such as O-PPy/PVA/EL, P-PPy/PVA/EL, Q-PPy/PVA/EL and R-PPy/PVA/EL, were fabricated by varying the concentration of the Fe^3+^ cations to polymerize different sizes of PPy nanoparticles to investigate the size-dependent electromechanical actuation performance. As the concentration of the Fe^3+^ cations was increased from O-PPy/PVA/EL to R-PPy/PVA/EL, the actuation performance of the actuator films decreased significantly. The high concentration of the Fe^3+^ cations grounded a strong electrostatic repulsion among the complex of PVA/Fe^3+^ cations, which might lead to the less control and conformational change and resulted in large PPy nanoparticles. At a low concentration of the Fe^3+^ cations, the deposition of the PPy nanoparticles on PVA involved in the polymerization reaction was sufficient. This led to the formation of fine polymer grains containing surface structure of the O-PPy/PVA/EL actuator film with smaller PPy nanoparticles. The results obtained with the composition of the O-PPy/PVA/EL actuator film showed an improved Young's modulus, ultimate tensile strength, tip-generated force, bending displacement and other electromechanical properties compared to that of P-PPy/PVA/EL, Q-PPy/PVA/EL and R-PPy/PVA/EL films. We developed a two finger-based micro-gripping device using O-PPy/PVA/EL films, and results show that this material composition can be used in dexterous handling devices in micro-robotic manipulation and artificial muscles applications. Therefore, the novel O-PPy/PVA/EL-based actuator had the potential to be used in biomimetic and micro robotic applications.

## Conflicts of interest

There are no conflicts to declare.

## Supplementary Material

RA-009-C9RA06900C-s001
