# Supplementary material for: Polypyrrole nanoparticles-based soft actuator for artificial muscle applications
Source: RSC Adv. 2019 Dec 2;9(68):39721–34. doi: 10.1039/c9ra06900c (PMC9076173; doi:10.1039/c9ra06900c)
Supplement: RA-009-C9RA06900C-s001 [file RA-009-C9RA06900C-s001.pdf]

Electronic Supplementary Material (ESI) for RSC Advances.
This journal is © The Royal Society of Chemistry 2019

Polypyrrole nanoparticles-based soft actuator for artificial muscle applications

Ajahar Khan[a], Khalid A. Alamry*[a], Ravi Kant Jain[b]

[a]Faculty of Science, Department of Chemistry, King Abdulaziz University, Jeddah 21589, Saudi Arabia

[b]Microrobotics Laboratory/Information Technology Group, CSIR- Central Mechanical Engineering Research Institute (CMERI), Durgapur 713209, India

Email: kaalamri@kau.edu.sa, arkhan.029@gmail.com

Supplementary Information

S.1 TEM

For acquiring further information about the internal structure of the fabricated soft actuator, TEM analysis was conducted and shown in Supplementary Fig. S1 (a-c). Supplementary Fig. S1 (a-c) clearly showed that PPy nanoparticles of 4.03-7.0 nm were uniformly rooted and has intimate contact with PVA matrices. As shown in Supplementary Fig. S1 (d), after deposition of PEDOT: PSS/SWNT/IL electrode film on the surface of PPy/PVA/EL based electrolyte membrane, the PEDOT: PSS and SWNT were stacked together to form a composite structure. It can be clearly seen that the electrode materials homogeneously intimate and covered the grey background of PPy/PVA/EL matrices of the fabricated soft actuator (Supplementary Fig. S1 (d)). The TEM analysis reveals the uniform distribution and intimate contact of the SWNT and PEDOT: PSS with PPy/PVA/EL polymer composite.

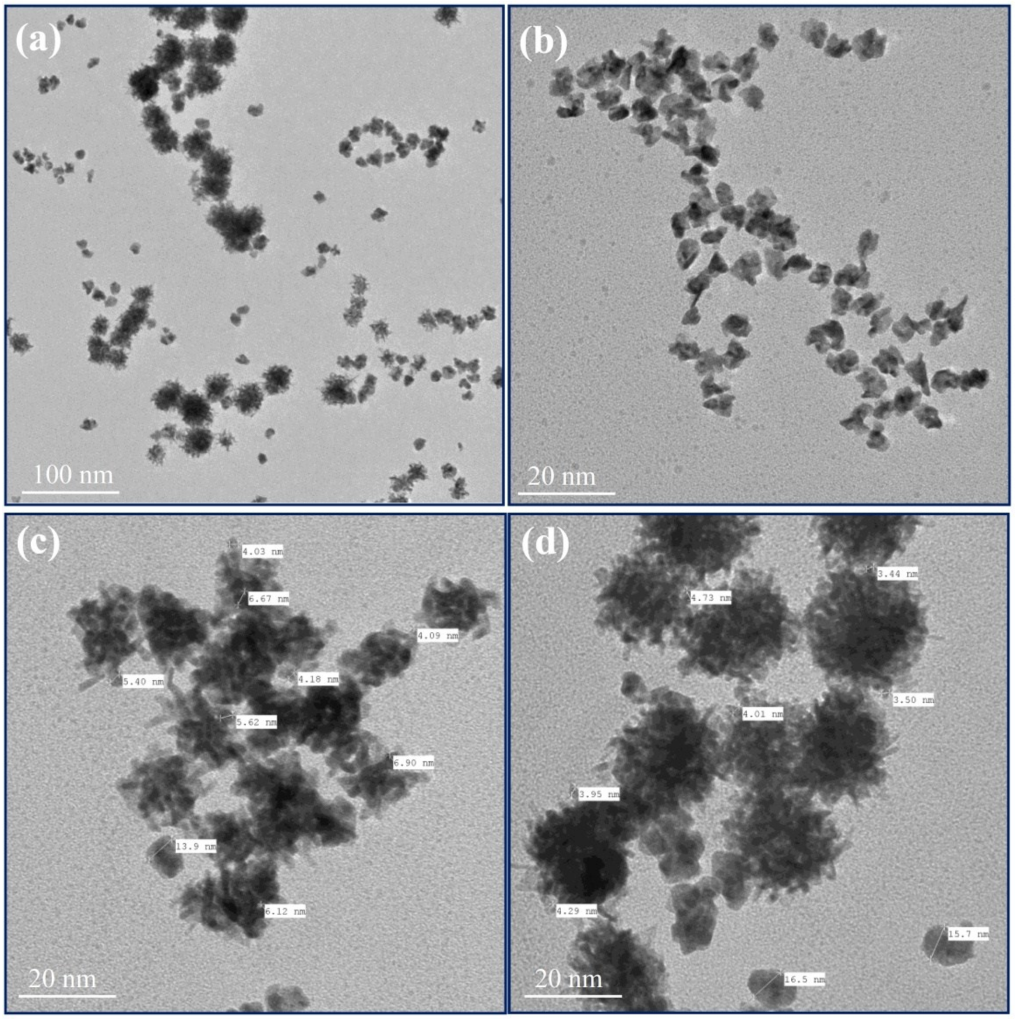

Supplementary Fig. S1 TEM images of the fabricated ionic polymer actuator films.

S.2 FTIR

FTIR analysis was conducted from 4000 to 400 cm^{-1}, and the results of the fabricated (a) O-PPy/PVA/EL, (b) P-PPy/PVA/EL, (c) Q-PPy/PVA/EL and (d) R-PPy/PVA/EL films are shown in Supplementary Fig. S2. Peaks observed at 3400 and 2940 cm^{-1}; were assigned due to the O-H stretching mode of hydroxyl groups (-OH), and asymmetric stretching of $-CH_2$ (Supplementary Fig. S2 (a-d)). The peaks appearing between 1000 and 1160 cm^{-1} were due to C-O stretching[1] (Supplementary Fig. S2 (b-d)). The peaks at 3440, 2360 and 1630 cm^{-1} are respectively assigned

due to -N-H and -CH stretching and N-H bending (Supplementary Fig. S2 (a-d). The peaks appeared at 1550 and 1460 cm^{-1} ascribed due to the anti-symmetric and symmetric stretching vibrations of C=C in PPy ring[2]. Other characteristic peaks of C-N (1306 cm^{-1}), C-C (1185 cm-1), N-H (1040 cm-1) and C-H (907, 780 and 675 cm^{-1}) bonds are also clearly observed[3], and these results confirm the successful formation of polypyrrole nanoparticles (Supplementary Fig. S2 (a-d)). As shown in Supplementary Fig. S2 (b), the absorption bands at 1215 and 1580 cm^{-1} could be assigned to the C-O stretch of the acid and C=C stretch of SWNT backbones, respectively[4]. The absorption peak at 1722 cm^{-1} confirms the existence of the carboxylic acid groups (Supplementary Fig. S2 (b))[4,5]. The absorption bands at 1574 and 1378 cm^{-1} suggest the formation of SWNT-$COO^{-}Na^{+}$ (Supplementary Fig. S2 (a))[6].

The characteristics peaks of PEDOT: PSS at 3700 cm^{-1} and 1852 cm^{-1} assigned due to O-H stretching and stretching frequency of C=S bond stretching mode of the sulfoxide groups (Supplementary Fig. S2 (b)) [7]. The absorption at ~1141cm^{-1} for C-O-S stretching vibration and ~1240 $cm^{-1}$for O-S-O asymmetric stretching vibration demonstrated that -SO_3H was present in the membrane actuator [8]. By the composition of PEDOT: PSS, PVA and SWNT, the intensity of several peaks were enhanced noticeably due to symmetric and asymmetric stretching bands of S=O which generally appear at the same frequency of C-O stretching [9].

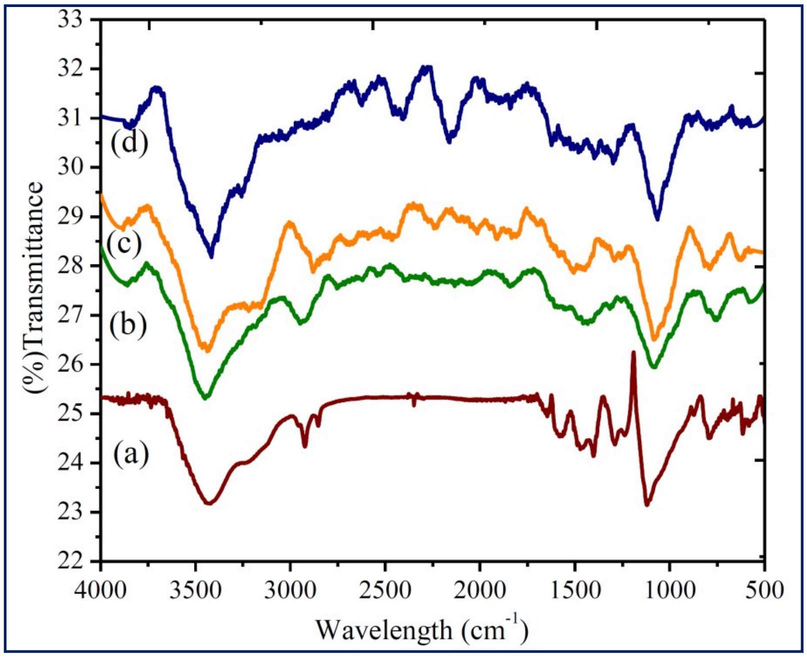

Supplementary Fig. S2 FT-IR spectra for (a) O-PPy/PVA/EL (b) P-PPy/PVA/EL, (c) Q-PPy/PVA/EL and (d) R-PPy/PVA/EL film actuators.

S.3 Ion exchange capacity (IEC)

The IEC of O-, P-, Q and R-PPy/PVA/EL actuator films were measured by a classical titration method. This method is used to measure the H^+ ions released by neutral salts to move through the polymer film. Firstly, the dried fabricated films (0.25 g) were cutted into very small pieces and treated with 1 M HNO_3 for 24 h at R.T., to change into H^+ form. Then the then the polymer films were washed with double distilled water till neutralization and dried at 60 ºC for 6 h. After that, the actuator films were stuffed into a glass column. Now, 1M aqueous solution of $NaNO_3$ as eluent was utilized to elute the protons totally from the column, with the flow rate of 0.5 ml min^{-1}. Then, the exchanged proton (H^+) of the solution was titrated with standard (0.1 M) NaOH solution using phenolphthalein indicator. Finally, the IEC was calculated using following equations:

$$\text{IEC} = \frac{Concentraion\ of\ NaOH\ \times Vol\ of\ NaOH}{Weight\ of\ dry\ membrane} \qquad \ldots\ldots\ldots\ldots\ldots (1)$$

S.4 Ionic or proton conductivity

Ionic or proton conductivity of the O-, P-, Q and R-PPy/PVA/EL films were measured by modular potentiostat/galvanostat (Autolab 302N) associated with an impedance analyser (FRA32M.X). A customary three-electrode framework including the O-, P-, Q and R-PPy/PVA/EL based actuator films were used as working electrode, Ag/AgCl as reference and a platinum wire counter electrode were utilized for PC investigation. The deliberate procedure was completed over a frequency range of 100 KHz under an AC annoyance of 100 mV. The proton conductivity (σ) of the O-, P-, Q and R-PPy/PVA/EL films were calculated using the following equation:

$$\sigma = \frac{L}{R \times A} \ldots\ldots\ldots\ldots 2$$

where σ stands for proton conductivity (S cm^{-1}), L for the thickness of the membrane (cm), R is the resistance (Ω), and A for cross-sectional area of the membrane (cm^2).

S.5 Voltage vs. tip generated force behavior

The tip generated force behavior for fabricated actuator films was also confirmed that the tip force was also proportional to the applied voltages like the tip displacement. The tip displacement and tip generated force both were found to be increasing with the increase of applied voltage (Supplementary Fig. S6). Therefore, large tip displacement reflects the larger generated force through the tip of the ionic polymer membrane. Thus, the results suggested that due to higher tip displacement of O-PPy/PVA/EL based actuator film, the generated force was also higher than the P-PPy/PVA/EL, Q-PPy/PVA/EL and R-PPy/PVA/EL actuator films.

Supplementary Figures

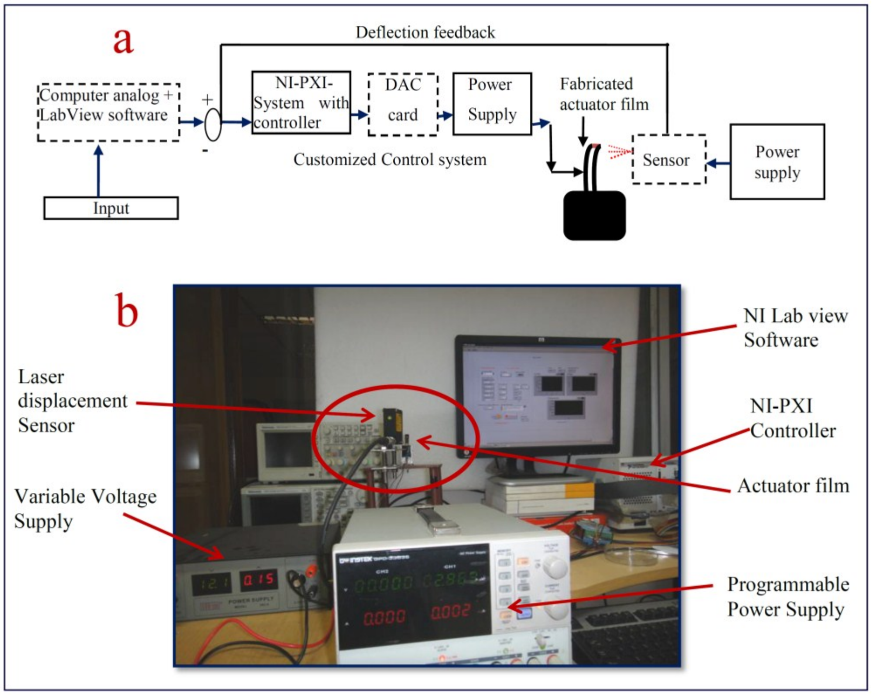

Supplementary Fig. S3 (a) Schematic representation and (b) actual view of experimental testing setup to analyses the tip displacement of the fabricated actuator films.

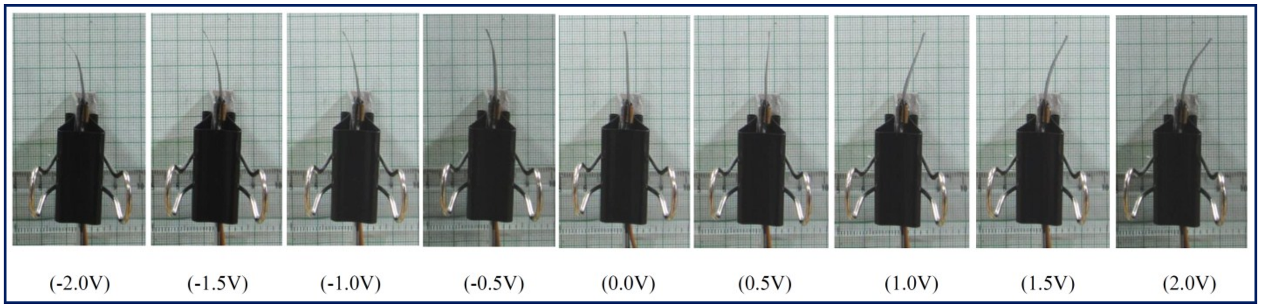

Supplementary Fig. S4 Successive tip deflection behavior of O-PPy/PVA/EL based actuator film.

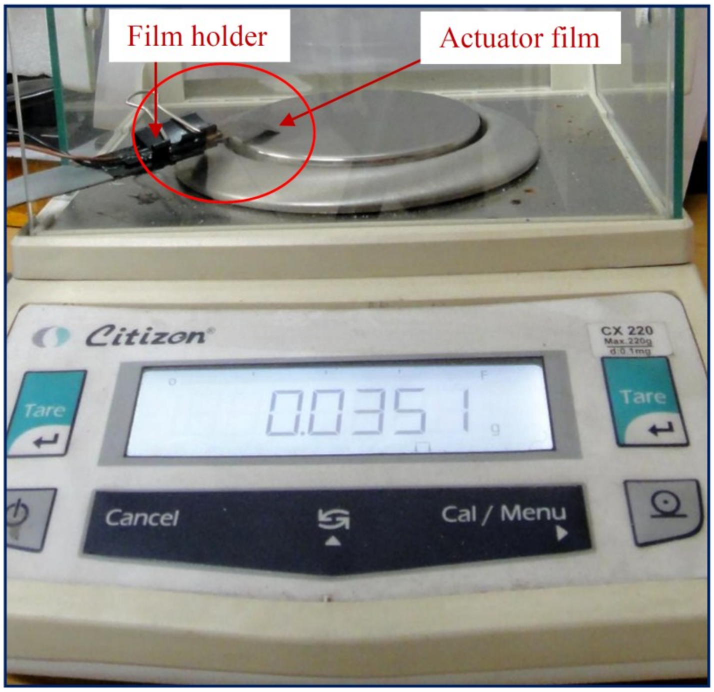

Supplementary Fig. S5 Load characterization testing setup for the fabricated PPy/PVA/EL based actuator film.

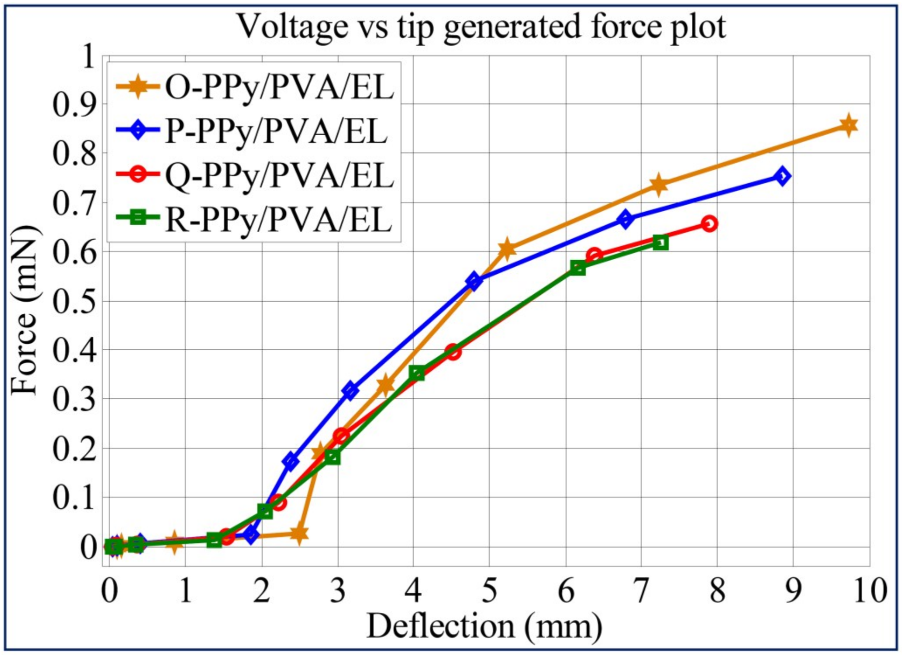

Supplementary Fig. S6 Force vs. deflection behavior for (a) O-PPy/PVA/EL, (b) P-PPy/PVA/EL, (c) Q-PPy/PVA/EL and (d) R-PPy/PVA/EL based actuator films.

Supplementary Tables

Supplementary Table S1 In-situ oxidative polymerization of PPy for the fabrication of actuator films with various diameters.

Composite Samples	Each composition in 10 mL DMW at 70 °C			
	**PVA (g)**	**Pyrrole (mL)**	**FeCl3 (g)**	**Remarks**
O-PPy/PVA	1.0	1.0	0.25	smooth film, cavity free
P-PPy/PVA	1.0	1.0	0.50	smooth film, small cavity
Q-PPy/PVA	1.0	1.0	0.75	porous film
R-PPy/PVA	1.0	1.0	1.0	porous film

Supplementary Table S2 Tensile properties of (a) O-PPy/PVA/EL, (b) P- PPy/PVA/EL, (c) Q-PPy/PVA/EL and (d) R- PPy/PVA/EL based actuator films.

Fabricated actuator films	Young's modulus (MPa)	Ultimate tensile strength (MPa)	Elongation at break (%)
O-PPy/PVA/EL	568.41	37.72	6.93
P- PPy/PVA/EL	529.19	36.19	7.95
Q- PPy/PVA/EL	440.97	34.14	8.98
R- PPy/PVA/EL	386.37	32.08	9.77

Supplementary Table S3 Experimental deflection data of O-PPy/PVA/EL ionic polymer actuator for trial 1.

Deflection (mm)	Voltage (V)								
	**0.0V**	**0.25V**	**0.50V**	**0.75V**	**1.00V**	**1.25V**	**1.50V**	**1.75V**	**2.00V**
d1	0.08	0.150	0.867	2.424	2.985	3.658	5.234	7.112	9.564
d2	0.07	0.143	0.958	2.654	2.895	3.568	5.421	7.245	9.664
d3	0.08	0.154	0.657	2.684	2.365	3.658	5.124	7.324	9.795
d4	0.12	0.196	0.856	2.465	2.647	3.687	5.145	7.124	9.695
d5	0.10	0.168	0.954	2.365	2.867	3.657	5.312	7.321	9.821
d6	0.09	0.156	0.784	2.354	2.876	3.568	5.134	7.234	9.853

Supplementary Table S4 Experimental deflection data of O-PPy/PVA/EL ionic polymer actuator for trial 2.

Deflection (mm)	Voltage (V)								
	0.0V	0.25V	0.50V	0.75V	1.00V	1.25V	1.50V	1.75V	2.00V
d1	0.083	0.338	1.461	5.907	3.964	7.344	6.742	7.130	9.609
d2	0.631	0.722	1.513	5.194	3.398	7.426	5.875	7.270	9.731
d3	0.672	0.466	4.207	4.117	3.116	5.662	8.310	7.505	9.953
d4	0.749	0.855	3.223	4.561	3.627	5.685	6.736	7.307	9.698
d5	0.161	0.299	3.199	4.006	4.571	5.036	6.299	7.431	9.829
d6	0.379	0.347	1.376	2.664	3.078	7.244	6.783	7.245	9.885

Supplementary Table S5 Experimental deflection data of O-PPy/PVA/EL ionic polymer actuator for trial 3.

Deflection (mm)	Voltage (V)								
	0.0V	0.25V	0.50V	0.75V	1.00V	1.25V	1.50V	1.75V	2.00V
d1	0.275	0.387	0.984	4.267	5.491	7.213	7.964	7.204	9.587
d2	0.518	0.458	4.689	6.522	5.623	7.236	5.969	7.330	9.667
d3	0.608	0.531	3.590	4.880	3.953	6.856	8.021	7.431	9.883
d4	0.284	0.259	2.818	4.558	4.123	4.083	5.573	7.230	9.754
d5	0.187	0.363	3.277	3.295	6.835	4.708	7.938	7.361	9.878
d6	0.310	0.751	1.737	4.317	3.027	4.915	7.119	7.317	9.960

Supplementary Table S6 Experimental deflection data of O-PPy/PVA/EL ionic polymer actuator for trial 4.

Deflection (mm)	Voltage (V)								
	0.0V	0.25V	0.50V	0.75V	1.00V	1.25V	1.50V	1.75V	2.00V
d1	0.434	0.520	3.513	4.159	6.321	6.293	5.904	7.247	9.642
d2	0.404	0.211	3.038	5.965	6.118	6.087	5.847	7.376	9.798
d3	0.122	0.734	4.561	3.018	2.607	4.829	6.618	7.331	9.854
d4	0.603	0.775	3.460	2.999	4.249	5.419	5.940	7.229	9.835
d5	0.130	0.680	4.165	3.060	4.981	3.719	7.276	7.359	9.864
d6	0.141	0.262	2.604	3.922	4.548	7.516	6.496	7.294	9.952

Supplementary Table S7 Experimental deflection data of P-PPy/PVA/EL ionic polymer actuator for trial 1.

Deflection (mm)	Voltage (V)								
	0.0V	0.25V	0.50V	0.75V	1.00V	1.25V	1.50V	1.75V	2.00V
d1	0.04	0.098	0.465	1.852	2.345	3.125	4.734	6.856	8.854
d2	0.05	0.095	0.356	1.735	2.468	3.135	4.832	6.756	8.954
d3	0.03	0.096	0.354	1.625	2.365	3.136	4.651	6.645	8.865
d4	0.06	0.089	0.354	1.985	2.352	3.232	4.822	6.865	8.756
d5	0.02	0.095	0.395	1.956	2.421	3.235	4.885	6.765	8.865
d6	0.04	0.098	0.452	1.986	2.352	3.135	4.885	6.864	8.894

Supplementary Table S8 Experimental deflection data of P-PPy/PVA/EL ionic polymer actuator for trial 2.

Deflection (mm)	Voltage (V)								
	0.0V	0.25V	0.50V	0.75V	1.00V	1.25V	1.50V	1.75V	2.00V
d1	0.555	0.569	2.948	4.202	4.737	4.827	7.303	6.925	8.872
d2	0.350	0.275	1.423	2.168	2.558	3.514	4.967	6.896	9.058
d3	0.182	0.403	3.671	5.272	4.076	5.544	4.927	6.767	8.998
d4	0.402	0.555	4.308	5.525	3.610	5.127	6.108	7.030	8.828
d5	0.405	0.296	3.334	5.247	3.070	6.036	7.021	6.855	8.880
d6	0.136	0.330	1.836	3.035	3.071	5.951	7.519	6.919	8.939

Supplementary Table S9 Experimental deflection data of P-PPy/PVA/EL ionic polymer actuator for trial 3.

Deflection (mm)	Voltage (V)								
	0.0V	0.25V	0.50V	0.75V	1.00V	1.25V	1.50V	1.75V	2.00V
d1	0.479	0.585	2.068	4.239	5.147	4.927	8.081	6.979	8.904
d2	0.590	0.160	3.091	2.803	3.931	3.214	6.583	6.871	9.056
d3	0.683	0.463	3.374	1.806	5.365	4.484	8.267	6.702	8.932
d4	0.741	0.460	2.472	5.060	3.960	4.960	6.415	6.898	8.883
d5	0.154	0.698	1.811	2.945	5.205	4.336	8.018	6.885	8.982
d6	0.137	0.437	1.063	3.788	5.220	3.937	6.501	7.009	8.919

Supplementary Table S10 Experimental deflection data of P-PPy/PVA/EL ionic polymer actuator for trial 4.

Deflection (mm)	Voltage (V)								
	0.0V	0.25V	0.50V	0.75V	1.00V	1.25V	1.50V	1.75V	2.00V
d1	0.525	0.492	1.465	5.463	2.413	5.364	6.719	6.863	8.946
d2	0.833	0.808	3.058	5.717	2.958	5.688	5.612	6.865	9.031
d3	0.447	0.551	0.692	4.743	5.862	3.265	5.105	6.693	8.886
d4	0.439	0.758	2.892	4.342	4.315	5.724	5.655	6.958	8.840
d5	0.654	0.679	3.074	5.719	5.846	4.704	5.479	6.885	9.012
d6	0.220	0.562	3.410	4.338	3.201	3.335	5.651	6.950	9.045

Supplementary Table S11 Experimental deflection data of Q-PPy/PVA/EL ionic polymer actuator for trial 1.

Deflection (mm)	Voltage (V)								
	0.0V	0.25V	0.50V	0.75V	1.00V	1.25V	1.50V	1.75V	2.00V
d1	0.05	0.086	0.435	1.657	2.432	3.024	4.564	6.546	7.824
d2	0.03	0.065	0.312	1.564	2.214	3.045	4.765	6.354	7.954
d3	0.04	0.075	0.332	1.457	2.246	3.043	4.324	6.235	7.836
d4	0.05	0.086	0.341	1.468	2.134	3.056	4.365	6.236	7.938
d5	0.04	0.075	0.361	1.467	2.135	3.074	4.465	6.452	7.868
d6	0.05	0.082	0.365	1.642	2.124	3.086	4.654	6.436	7.989

Supplementary Table S12 Experimental deflection data of Q-PPy/PVA/EL ionic polymer actuator for trial 2.

Deflection (mm)	Voltage (V)								
	0.0V	0.25V	0.50V	0.75V	1.00V	1.25V	1.50V	1.75V	2.00V
d1	0.053	0.274	1.029	5.140	3.411	6.710	6.072	6.564	7.869
d2	0.591	0.644	0.867	4.104	2.717	6.903	5.219	6.379	8.021
d3	0.632	0.387	3.882	2.890	2.997	5.047	7.510	6.416	7.994
d4	0.679	0.745	2.708	3.564	3.114	5.054	5.956	6.419	7.941
d5	0.101	0.206	2.606	3.108	3.839	4.453	5.452	6.562	7.876
d6	0.339	0.273	0.957	1.952	2.326	6.762	6.303	6.447	8.021

Supplementary Table S13 Experimental deflection data of Q-PPy/PVA/EL ionic polymer actuator for trial 3.

Deflection (mm)	Voltage (V)								
	0.0V	0.25V	0.50V	0.75V	1.00V	1.25V	1.50V	1.75V	2.00V
d1	0.245	0.323	0.552	3.500	4.938	6.579	7.294	6.638	7.847
d2	0.478	0.380	4.043	5.432	4.942	6.713	5.313	6.439	7.957
d3	0.568	0.452	3.265	3.653	3.834	6.241	7.221	6.342	7.924
d4	0.214	0.149	2.303	3.561	3.61	3.452	4.793	6.342	7.997
d5	0.127	0.270	2.684	2.397	6.103	4.125	7.091	6.491	7.925
d6	0.270	0.677	1.318	3.605	2.275	4.433	6.639	6.519	8.096

Supplementary Table S14 Experimental deflection data of Q-PPy/PVA/EL ionic polymer actuator for trial 4.

Deflection (mm)	Voltage (V)								
	0.0V	0.25V	0.50V	0.75V	1.00V	1.25V	1.50V	1.75V	2.00V
d1	0.404	0.456	3.081	3.392	5.768	5.659	5.234	6.681	7.902
d2	0.364	0.133	2.392	4.875	5.437	5.564	5.191	6.485	8.088
d3	0.082	0.655	4.236	1.791	2.488	4.214	5.818	6.242	7.895
d4	0.533	0.665	2.945	2.002	3.736	4.788	5.160	6.341	8.078
d5	0.070	0.587	3.572	2.162	4.249	3.136	6.429	6.490	7.911
d6	0.100	0.188	2.185	3.210	3.796	7.034	6.016	6.496	8.088

Supplementary Table S15 Experimental deflection data of R-PPy/PVA/EL ionic polymer actuator for trial 1.

Deflection (mm)	Voltage (V)								
	0.0V	0.25V	0.50V	0.75V	1.00V	1.25V	1.50V	1.75V	2.00V
d1	0.02	0.085	0.385	1.452	2.124	2.985	4.023	6.124	7.191
d2	0.04	0.061	0.411	1.465	2.021	2.956	4.058	6.138	7.341
d3	0.05	0.064	0.321	1.421	2.042	2.864	4.035	6.165	7.262
d4	0.05	0.067	0.312	1.341	2.042	2.887	4.064	6.182	7.284
d5	0.06	0.081	0.361	1.312	2.008	2.984	4.032	6.175	7.265
d6	0.04	0.061	0.321	1.314	2.038	2.968	4.056	6.168	7.235

Supplementary Table S16 Experimental deflection data of R-PPy/PVA/EL ionic polymer actuator for trial 2.

Deflection (mm)	Voltage (V)								
	**0.0V**	**0.25V**	**0.50V**	**0.75V**	**1.00V**	**1.25V**	**1.50V**	**1.75V**	**2.00V**
d1	0.023	0.273	0.979	4.935	3.103	6.671	5.531	6.142	7.236
d2	0.601	0.640	0.966	4.005	2.524	6.814	4.512	6.163	7.408
d3	0.642	0.376	3.871	2.854	2.793	4.868	7.221	6.346	7.42
d4	0.679	0.726	2.679	3.437	3.022	4.885	5.655	6.365	7.287
d5	0.121	0.212	2.606	2.953	3.712	4.363	5.019	6.285	7.273
d6	0.329	0.252	0.913	1.624	2.240	6.644	5.705	6.179	7.267

Supplementary Table S17 Experimental deflection data of R-PPy/PVA/EL ionic polymer actuator for trial 3.

Deflection (mm)	Voltage (V)								
	**0.0V**	**0.25V**	**0.50V**	**0.75V**	**1.00V**	**1.25V**	**1.50V**	**1.75V**	**2.00V**
d1	0.215	0.322	0.502	3.295	4.630	6.540	6.753	6.216	7.214
d2	0.488	0.376	4.142	5.333	4.749	6.624	4.606	6.223	7.344
d3	0.578	0.441	3.254	3.617	3.630	6.062	6.932	6.272	7.350
d4	0.214	0.130	2.274	3.434	3.518	3.283	4.492	6.288	7.343
d5	0.147	0.276	2.684	2.242	5.976	4.035	6.658	6.214	7.322
d6	0.260	0.656	1.274	3.277	2.189	4.315	6.041	6.251	7.342

Supplementary Table S18 Experimental deflection data of R-PPy/PVA/EL ionic polymer actuator for trial 4.

Deflection (mm)	Voltage (V)								
	**0.0V**	**0.25V**	**0.50V**	**0.75V**	**1.00V**	**1.25V**	**1.50V**	**1.75V**	**2.00V**
d1	0.374	0.455	3.031	3.187	5.460	5.620	4.693	6.259	7.269
d2	0.374	0.129	2.491	4.776	5.244	5.475	4.484	6.269	7.475
d3	0.092	0.644	4.225	1.755	2.284	4.035	5.529	6.172	7.321
d4	0.533	0.646	2.916	1.875	3.644	4.619	4.859	6.287	7.424
d5	0.090	0.593	3.572	2.007	4.122	3.046	5.996	6.213	7.308
d6	0.090	0.167	2.141	2.882	3.710	6.916	5.418	6.228	7.334

Supplementary Table S19 Calculated tip generated force data for O-PPy/PVA/EL ionic polymer film actuator.

Force (mN)	Voltage (V)								
	0 V	0.25	0.50	0.75V	1.00	1.25V	1.5V	1.75V	2.0V
F1	0	0.0012	0.0065	0.0214	0.1352	0.3245	0.5124	0.7846	0.8695
F2	0	0.0013	0.0075	0.0234	0.1542	0.2315	0.6524	0.7458	0.8554
F3	0	0.0010	0.0068	0.0310	0.2103	0.3415	0.5628	0.7628	0.8695
F4	0	0.0015	0.0095	0.0321	0.2151	0.3524	0.6514	0.7451	0.8597
F5	0	0.0023	0.0082	0.0216	0.1985	0.3954	0.6524	0.6754	0.7985

F6	0	0.0024	0.0067	0.0268	0.2121	0.3214	0.5986	0.6985	0.8865
								Operating voltage	2.0 V
								Mean	0.8565
								Standard deviation	0.0303 mN

Supplementary Table S20 Calculated tip generated force data for P-PPy/PVA/EL ionic polymer film actuator.

Force (mN)	Voltage (V)								
	0 V	0.25	0.50	0.75V	1.00	1.25V	1.5V	1.75V	2.0V
F1	0	0.0008	0.0052	0.0211	0.1265	0.3168	0.4954	0.7021	0.7986
F2	0	0.0010	0.0065	0.0216	0.1365	0.3134	0.5102	0.6897	0.8031
F3	0	0.0008	0.0049	0.0264	0.1985	0.3245	0.5874	0.7105	0.7402
F4	0	0.0011	0.0065	0.0254	0.1895	0.3314	0.5985	0.6895	0.7291
F5	0	0.0013	0.0071	0.0221	0.1867	0.3104	0.5357	0.5986	0.7184
F6	0	0.0016	0.0062	0.0251	0.1987	0.3025	0.5124	0.6058	0.7362
								Operating voltage	2.0 V
								Mean	0.7542
								Standard deviation	0.0368 mN

Supplementary Table S21 Calculated tip generated force data for Q-PPy/PVA/EL ionic polymer membrane actuator.

Force (mN)	Voltage (V)								
	0 V	0.25	0.50	0.75V	1.00	1.25V	1.5V	1.75V	2.0V
F1	0	0.0004	0.0041	0.0186	0.0896	0.2485	0.3678	0.6127	0.6157
F2	0	0.0007	0.0042	0.0184	0.0958	0.2157	0.4312	0.5968	0.6589
F3	0	0.0010	0.0039	0.0195	0.0865	0.2035	0.3985	0.5647	0.6984
F4	0	0.0006	0.0051	0.0187	0.0798	0.2087	0.3895	0.5684	0.7023
F5	0	0.0010	0.0058	0.0179	0.0957	0.2114	0.4215	0.6101	0.6412
F6	0	0.0009	0.0054	0.0194	0.0934	0.2647	0.3689	0.5897	0.6214
								Operating voltage	2.0 V
								Mean	0.6563
								Standard deviation	0.0373 mN

Supplementary Table S22 Calculated tip generated force data for R-PPy/PVA/EL ionic polymer film actuator.

Force (mN)	Voltage (V)								
	0 V	0.25	0.50	0.75V	1.00	1.25V	1.5V	1.75V	2.0V
F1	0	0.0005	0.0031	0.0125	0.0845	0.1986	0.3841	0.5486	0.5831
F2	0	0.0002	0.0038	0.0165	0.0761	0.1957	0.3854	0.5112	0.5767
F3	0	0.0004	0.0028	0.0121	0.0714	0.1864	0.3025	0.5864	0.6241
F4	0	0.0002	0.0043	0.0109	0.0689	0.1457	0.3695	0.6114	0.6741
F5	0	0.0005	0.0034	0.0135	0.0642	0.1684	0.3785	0.5762	0.6054

F6	0	0.0002	0.0042	0.0164	0.0674	0.2013	0.2989	0.5691	0.6414
Operating voltage									2.0 V
Mean									0.6174
Standard deviation									0.0369 mN

References

1 M. Y. Kariduraganavar, S. S. Kulkarni and A. A. Kittur, *J. Memb. Sci.*, 2005, **246**, 83–93.

2 Y. Jia, P. Xiao, H. He, J. Yao, F. Liu, Z. Wang and Y. Li, *Appl. Surf. Sci.*, 2012, **258**, 6627–6631.

3 X. Zhang, J. Wang, J. Liu, J. Wu, H. Chen and H. Bi, *Carbon N. Y.*, 2017, **115**, 134–146.

4 L. Liu, Y. Qin, Z.-X. Guo and D. Zhu, *Carbon N. Y.*, 2003, **41**, 331–335.

5 J. Zhang, H. Zou, Q. Qing, Y. Yang, Q. Li, Z. Liu, X. Guo and Z. Du, *J. Phys. Chem. B*, 2003, **107**, 3712–3718.

6 M. A. Hamon, J. Chen, H. Hu, Y. Chen, M. E. Itkis, A. M. Rao, P. C. Eklund and R. C. Haddon, *Adv. Mater.*, 1999, **11**, 834–840.

7 A. Pasha, A. S. Roy, M. V. Murugendrappa, O. A. Al-Hartomy and S. Khasim, *J. Mater. Sci. Mater. Electron.*, 2016, **27**, 8332–8339.

8 T. Yang, *J. Memb. Sci.*, 2009, **342**, 221–226.

9 C. Chanthad and J. Wootthikanokkhan, *J. Appl. Polym. Sci.*, 2006, **101**, 1931–1936.
